# Incorporation of methotrexate into coconut oil nanoemulsion potentiates its antiproliferation activity and attenuates its oxidative stress

**DOI:** 10.1080/10717544.2020.1736209

**Published:** 2020-03-05

**Authors:** Mayson H. Alkhatib, Shaza A. Alyamani, Faiza Abdu

**Affiliations:** a Department of Biochemistry, King Abdulaziz University, Jeddah, Saudi Arabia;; b Department of Biological Sciences, Faculty of Science, King Abdulaziz University, Jeddah, Saudi Arabia

**Keywords:** Chemotherapeutic agents, A549 non-small cell lung cancer cells, Ehrlich ascites carcinoma, essential oils, nanocarrier

## Abstract

Methotrexate (MTX), a chemotherapeutic agent, has limited clinical applications due to its pulmonary and neurotoxicity. The antineoplastic activity of MTX-NE COCO, which is MTX formulated in coconut oil nanoemulsion (NE), was evaluated in A549 non-small cell lung cancer cells while its adverse side effects on the oxidative stress of the lung and brain were assessed in mice. The *z*-average diameter for the dispersed nanodroplet of MTX-NE COCO (79.74 ± 3.49 nm) was considerably greater than the free-NE COCO (64.80 ± 3.34 nm). In contrast, the magnitude of the negative *z*-potential of MTX-NE COCO (3.00 ± 0.69 mV) was markedly less than that of free-NE COCO (8.20 ± 0.76 mV). The minimum inhibitory concentration (IC_50_) of MTX-NE COCO (18 ± 1.8 µM) was less than the IC_50_ of free MTX (32 ± 1.2 µM) by around twofold. The *in vivo* evaluation of the MTX-NE COCO treatment revealed that the antioxidant enzymes activities of the brain and lung tissues, catalase, superoxide dismutase, and glutathione reductase, were relatively raised while the malondialdehyde amount was diminished when compared to the free MTX treatment. In conclusion, combining MTX with coconut oil in a NE had improved its efficacy while ameliorating its oxidative stress effect on the brain and lungs.

## 1. Introduction

Lung cancer, which is caused mainly by the abnormal growth of either the non-small or small lung cancer cells, is increasing rapidly worldwide (Alghamdi et al., 2018; Okuyama, 2018). In spite of the availability of the chemotherapeutic agents that eliminate the growth of the cancer cells, their severe toxic effects on the normal cells restrain their clinical applications (Anderson et al., 2009). In particular, the most important problem with the chemotherapeutic agents is drug resistance which results in enhancing their toxicity and reducing their efficacies (Howard et al., 2016).

Methotrexate (4-amino-4-deoxy-N^10^-methylpteroylglutamic acid), MTX, a weak bicarboxylic acid and hydrophilic drug, is commonly used as a treatment for some types of cancer, including brain, breast, ovaries, and leukemia. MTX is an antifolate belonging to the antimetabolite class of antineoplastic agents. It is a cell cycle-specific chemotherapeutic agent that acts on S-phase and inhibits DNA synthesis (Hashkes et al., 2014; Levêque et al., 2017). MTX, as a high-dose, could cause many and severe side effects. In particular, MTX can induce acute and chronic neurotoxicity (Watanabe et al., 2018). MTX is pulmonary toxic since it may cause alveolitis and lung fibroblasts (Howard et al., 2016; Saygin et al., 2016). MTX, loaded in layered double hydroxide nanoparticle, had exhibited a great antitumor activity against human osteosarcoma-bearing mice with reduced adverse side effects (Choi et al., 2013). It had been also reported that the efficacy of MTX, incorporated into a lipid–polymer hybrid nanoparticle, was improved due to its increased internalization into the cancer cell (Garg et al., 2017).

Recently, coconut oil is attracting the pharmaceutical industry because of its great nutritional impact on health (DebMandal & Mandal, 2011; Boateng et al., 2016). Coconut oil is a major source of lauric acid which is a middle chain fatty acid that is absorbed in the small intestine without undergoing degradation. It has protective properties against heart diseases, diabetes, cancer, and infectious diseases. The active components of coconut oil play a role as antioxidant and antitumor which was found to cause a reduction in A549 non-small cell lung cancer cell growth (Famurewa et al., 2018).

In order to assure complete mixing between the coconut oil and MTX in the present study, a suspension system, consisting of a surfactant, co-surfactant, and water was utilized to solubilize the coconut oil and MTX. The formulation of nanoemulsion (NE) as a nano-colloidal system has the potential to attenuate the drug’s bioavailability and efficacy by reducing the required dosage which may result in diminishing the drug’s toxicity (Shakeel et al., 2008; Jaiswal et al., 2015; Aboalnaja et al., 2016). Nanoemulsions are heterogeneous systems produced from specific arrangements of the oil and water with the means of surfactants/co-surfactants at percentages that do not exceed 10%. NEs have a droplet size of less than 100 nm and can be formed by energy input (heating and mixing) (Shakeel et al., 2008; Aboalnaja et al., 2016). As drug delivery systems, NEs are very attractive due to the small droplet size that allows them to deposit uniformly on substrates which result in possessing stability against sedimentation (Jaiswal et al., 2015; Aboalnaja et al., 2016). The main objectives of this study were to *in vitro* and *in vivo* examine the antitumor activity and cytotoxicity of MTX loaded in coconut oil NE.

## 2. Materials

### 2.1. Chemicals

Coconut oil was obtained from Abazer for Natural Oils (Jeddah, Saudi Arabia). MTX was purchased from Al-Foad Pharmacy (Cairo, Egypt). All of the tissue culture reagents and supplements were procured from Sigma Aldrich (St. Louis, MO).

### 2.2. Cell lines and animals

A549 non-small cell lung cancer and EAC cell lines were obtained from the Tissue Culture Unit at King Fahd Center for Medical Research (Jeddah, Saudi Arabia). Fifty mice were preserved in accordance with King Abdulaziz University’s policy and the International Ethical Guidelines on the Care and Use of Laboratory Animals (National Research Council, 2011). The ethical approval was obtained from the research ethics committee in the Faculty of Medicine at King Abdulaziz University (1-18-01-009-0013).

## 3. Methods

### 3.1. Preparation of coconut oil nanoemulsions

The drug-free coconut oil nanoemulsion (free-NE COCO) was prepared by mixing different weight percentages of 1.86 coconut oil, 3.72 Tween 80, 1.40 Span 20 and 93.02 distilled water. In particular, the Tween 80 and Span 20 were blended followed by adding dropwise the warm distilled water with vortexing and an incessant mixing until the milky mixture was formed. After that, the coconut oil was added slowly to the heated mixture (∼70 °C). The resulted emulsion was exposed to heating-cooling cycles continuously along with vortexing for 3–4 hours until a clear and transparent solution gets formed. The stock solution of 110 μM of MTX-NE COCO was produced by adding directly the MTX into the free-NE COCO whereas the stock 110 μM of MTX solution was produced by dissolving the MTX in the distilled water.

### 3.2. Physical characterization of coconut oil nanoemulsions

The nanodroplets diameter and charges of free-NE COCO and (1 mg/ml) MTX-NE COCO, expressed as the *z*-average diameter and zeta potential, respectively, were determined using Zetasizer Nano ZS (Malvern Instruments, Malvern, UK).

### 3.3. *In vitro* evaluation of the antitumor activity of coconut oil nanoemulsions

#### 3.3.1. Cell culture

A549 cells were grown in a 25 cm^2^ tissue culture flask, containing 10 ml of Dulbecco’s modified Eagle medium (DMEM) supplemented with 1% (v/v) penicillin–streptomycin antibiotic and 10% (v/v) fetal bovine serum albumin. After 24 h incubation in 95% air and 5% humidified CO_2_ incubator at 37 °C, the culture medium was discarded and changed at 48 h intervals. Cells were fed until confluence. Confluent cells were washed with 2 ml of phosphate-buffered saline, collected with 2 ml of trypsin and kept in the incubator at 37 °C.

#### 3.3.2. Cell growth inhibition assay

A549 cell line was cultured as described by Alkhatib & Alkhayyal (2016). In brief, each well-containing 100 µl of growth medium in a 96-well, flat-bottomed tissue culture plate was seeded with 5000 tested cells and incubated for 24 h at 37 °C in a humidified 5% CO_2_. After that, cells, treated with 100 μl of the desired concentration of free MTX, free-NE COCO, and MTX-NE COCO, were re-incubated for 24 h at 37 °C in a humidified 5% CO_2_ incubator. Following incubation, a 5 μl of the yellow MTT reagent was subjected to each well and kept for 4 h at 37 °C in a humidified 5% CO_2_ incubator. After that, the supernatant was removed and 100 μl of the dimethyl sulfoxide solution was added to each well and incubated for 10 min at 37 °C in a humidified 5% CO_2_ incubator. The absorbance of each well was measured at 570 nm using ELISA plate reader (BioTek, Winooski, VT). The cytotoxicity of the drug formula on the cells was detected according to the percentages of cell viability calculated by dividing the absorbance of the sample by the absorbance of the control and then multiplying by 100.

#### 3.3.3. Apoptosis detection method

The apoptosis effects of the tested formulas of the free MTX, free-NE COCO, and MTX-NE COCO were evaluated at their IC_50_ concentrations by two methods of staining for the A549 cells as described by Alkhatib et al. (2017). First, 5 × 10^4^/well of the untreated (control) and treated cells were stained with Coomassie Brilliant Blue to observe their morphological changes using a phase-contrast inverted light microscope (1 × 17 Olympus, Tokyo, Japan). Second, 5 × 10^4^/well of the untreated (control) and treated cells were stained with 9 nM of DAPI which is a fluorescent dye that gets attached to the A–T base pair of the dsDNA and therefore it reveals the nuclear changes in the cells under the fluorescent microscope (Leica DMI6000 B, Wetzlar, Germany).

### 3.4. *In vivo* antitumor activity of drug formulations

#### 3.4.1. Experimental design

The 50 mice were split into five groups at which each group had 10 mice as follows:

*Group I*: Untreated mice and served as the control (–ve);
*Group II*: Untreated EAC-bearing mice and served as the control (+ve);
*Group III* (free MTX): Received one dose of 20 mg of free MTX/kg of mouse/0.2 ml of distilled water (Abdel-Daim et al., 2017);
*Group IV* (free-NE COCO): Received one dose of 0.2 ml of free-NE COCO/mouse;
*Group V* (MTX-NE COCO): Received one dose of 20 mg of free MTX/kg of mouse/0.2 ml of free-NE COCO (Abdel-Daim et al., 2017).

It should be noted that groups II–V were injected intraperitoneally with 2.5 × 10^6^ EAC cells. The desired treatments of groups II–V were administered after 48 h of EAC inoculation once a week as adopted by Alkhatib et al. (2017). On the 7th day, the body weight of each mouse was taken and the mice from each group were sacrificed after fasting for 12 h. The organs were collected for the histological study. The organ weight ratios were calculated by dividing the post-sacrifice organ weight by the pre-sacrifice body weight of the same animal. For the antioxidant assays, small parts of the excised lung and brain organs were resected and rinsed in ice-cold normal saline.

#### 3.4.2. Oxidative stress assessment

The small parts of the brain and lung tissues were homogenized according to a procedure explained by AlMotwaa et al. (2019). The antioxidant status was identified by measuring the amount of lipid peroxide (malondialdehyde, MDA) and the activities of the superoxide dismutase (SOD), catalase (CAT), and glutathione reductase (GR). The commercial assays used for the examination of the oxidative stress were procured from Bio-diagnostic Lab for Diagnostic and Research Reagents (Cairo, Egypt).

#### 3.4.3. Histology examination

The brain and lung tissues were fixed at 10% formalin, treated, and microscopically examined as described elsewhere (AlMotwaa et al., 2019).

### 3.5. Statistical analysis

Statistical analysis was performed with one-way analysis of variance (ANOVA) test using the MegaStat Excel (version 10.3, Butler University, Indianapolis, IN). The significant variations between the groups were considered when *p* value <.05.

## 4. Results

### 4.1. Zetasizer measurements for NE characterization

The physical characteristics of the produced NE formulas are summarized in Table 1. A remarkable increase in the nanodroplet *z*-average diameter of the MTX-NE COCO formula was revealed when compared to the free-NE COCO formula (.001 < *p* ≤  .01). In contrast, the magnitude of the negative *z*-potential of the MTX-NE COCO formula was considerably less than the free-NE COCO formula (.001 < *p* ≤  .01). Interestingly, the polydispersity indices (PDIs) of the *z*-average diameters and *z*-potentials for both formulas were very small, indicating very limited variations among the droplet sizes and charges of the individual formula.

**Table 1.** The physical characteristics of the NE formulations measured by the Zetasizer.

Formulation	*z*-Average diameter (nm)	PDI	Zeta potential (mV)	PDI
Free-NECOCO	64.80 ± 3.34	0.052	−8.20 ± 0.76	0.093
MTX-NECOCO	79.74 ± 3.49	0.044	−3.00 ± 0.69	0.230
*p* Value	.006**		.009**	

PDI (polydispersity index = mean/SD). Data were expressed as mean ± standard deviation (SD) for three determinations.

** .001<*p* < .01.

### 4.2. *In vitro* evaluation of the antineoplastic activity of drug formulations

#### 4.2.1. MTT assay for cytotoxicity screening

**Figure 1.**
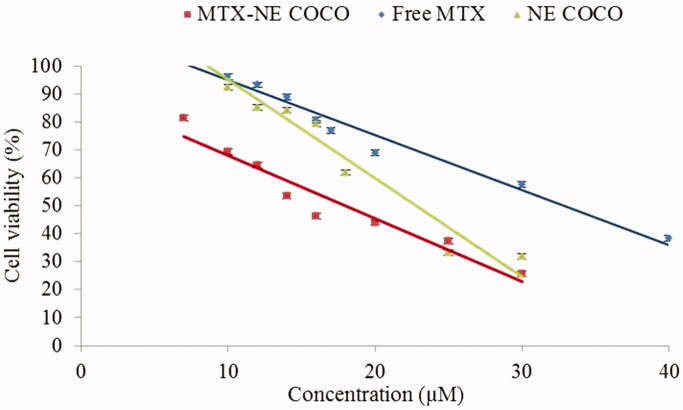
The percentage of A549 cell viabilities was determined by MTT assay after 24 h drug exposure with the desired concentrations of the tested formula. Error bars represent the standard deviation for *n* = 3.

**Figure 2.**
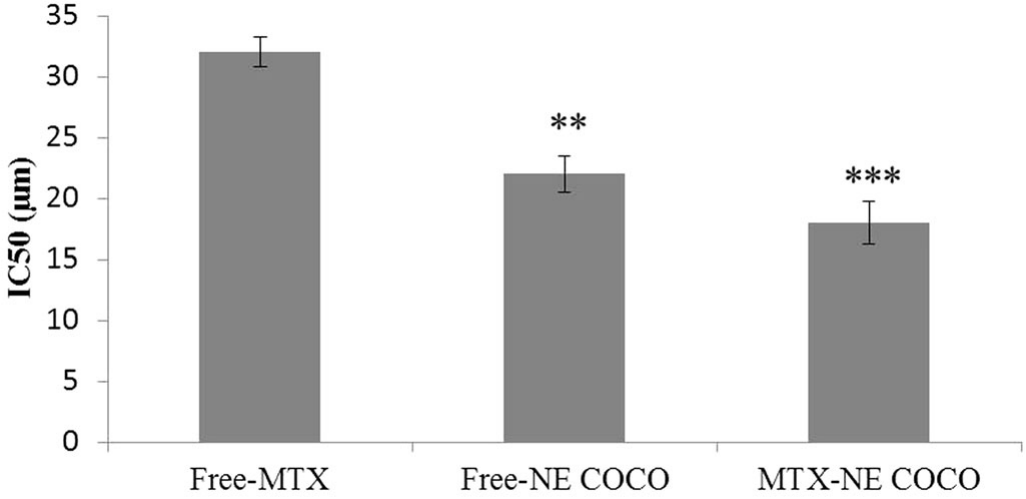
A graph represents the IC_50_ (µM) of the tested formulas when subjected to the A549 non-small cell lung cancer cells. The level of variations between the IC_50_ of free-MTX and the other NE formulas were expressed as highly (**.001<*p* < .01) and very highly (***p < .001) significant difference.

As clearly displayed in Figure 1, there were linear decreases in the percentages of cell viabilities as the concentration of the desired drug formula increased. MTX-NE COCO formula has caused the maximum reduction in the A549 cell viabilities when compared to the free MTX at all matched concentrations (*p* <  .001). According to Figure 2, the IC_50_ of MTX-NE COCO formula (18.00 ± 1.80) µM and free-NE COCO IC_50_ (22.00 ± 1.50) µM were markedly less than the IC_50_ of free MTX (32.00 ± 1.20) µM. It should be noted the IC_50_ of MTX-NE COCO formula was less than the IC_50_ of free MTX by around twofold (*p* <  .001).

#### 4.2.2. Light microscopy and DAPI stain for apoptosis detection

**Figure 3.**
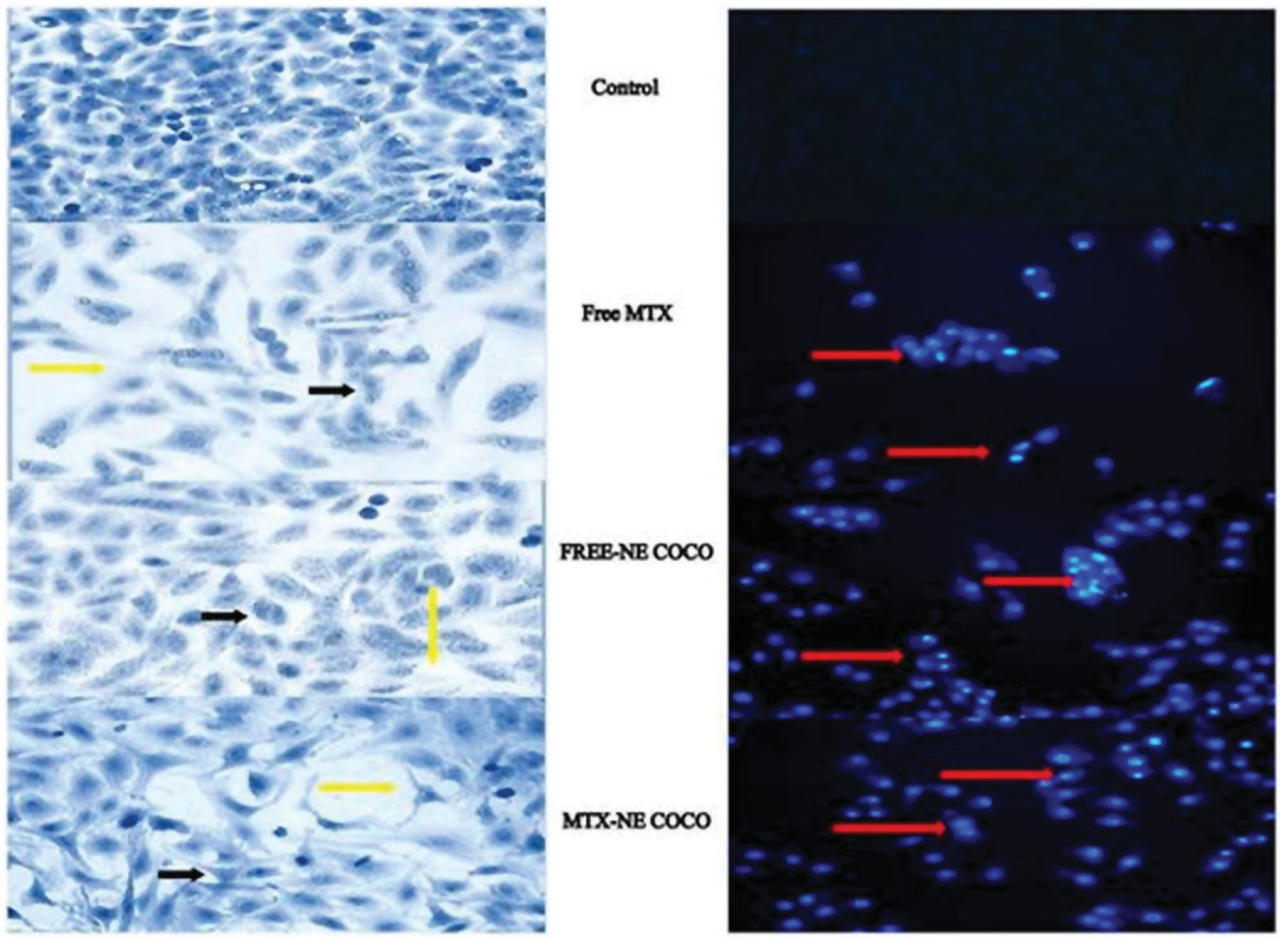
(A) Light microscopy images of A549 non-small cell lung cancer cells treated for 24 h at the IC_50_ of free MTX, free-NE COCO, and MTX-NE COCO. Signs of apoptosis are represented by the black arrows (chromatin condensation) and yellow arrows (intercellular space). Images were magnified at ×40. (B) Fluorescence microscopy images of DAPI stained A549 non-small cell lung cancer cells treated for 24 h at the IC_50_ of free MTX, free-NE COCO, and MTX-NE COCO. The red arrows point at the altered nuclei (chromatin condensation). Images were magnified at ×20.

The effects of the tested drug formulas at their IC_50_’s on the morphologies of the A549 cells were exhibited in Figure 3. The signs of apoptosis were seen in all of the light microscopy images of the treated cells which included the alterations in the cell’s shapes, the appearance of the condensed chromatin and the enhancement of the spaces between the cells. Additionally, it was observed by the fluorescence microscopy that the nuclei of the DAPI stained cells which were subjected to the tested formulas had fluoresced and get enlarged.

### 4.3. *In vivo* evaluation of the drug formulation’s side effects

#### 4.3.1. Brain tissue analysis

**Figure 4.**
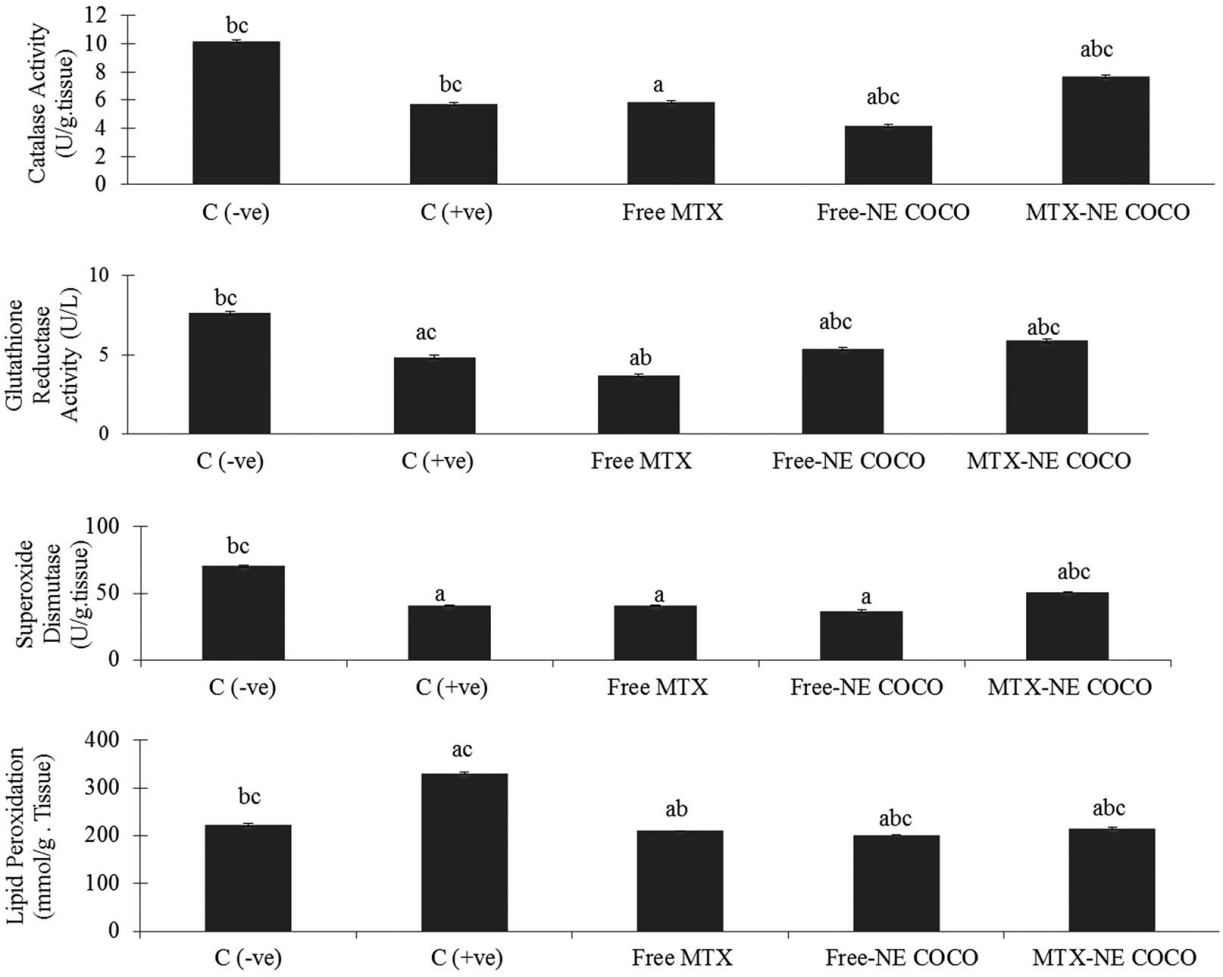
ROS values of brain tissue homogenates for the experimental groups. The superscripts (a, b, c) display the statistical significant variations between the desired group and C (–ve), C (+ve), and free MTX, respectively (*p* < .05).

*Oxidative stress analysis*. According to the antioxidant status analysis of the tested groups shown in Figure 4, the activities of CAT, SOD, and GR for both of the C (–ve) and MTX-NE COCO groups were markedly greater than that of C (+ve), free MTX, and free-NE COCO groups (*p* <  .05). On the other hand, the MDA levels were enhanced only in C (+ve) group when compared to the other tested groups (*p* <  .05).

**Figure 5.**
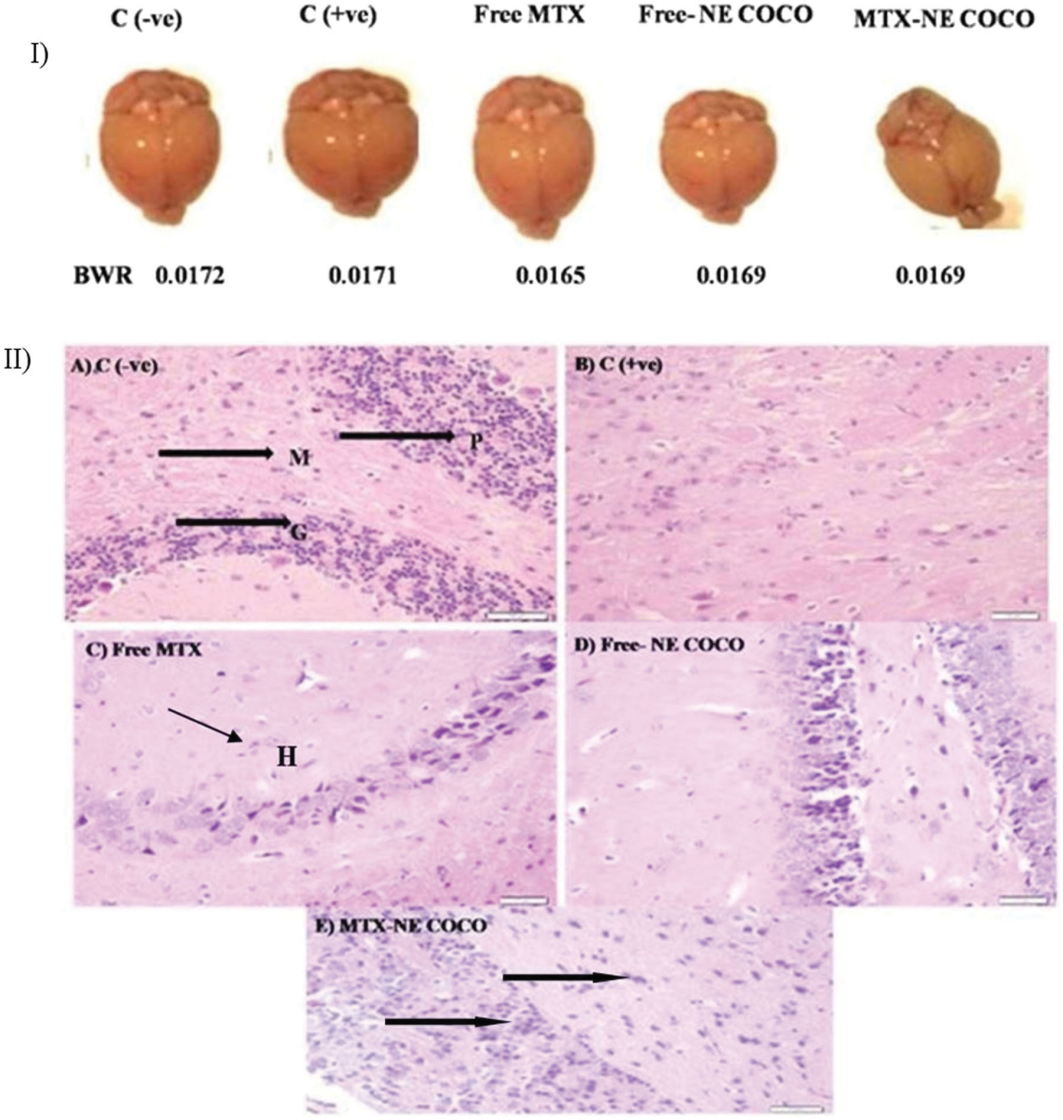
(I) Photomicrographs of the resected brain for the tested mice and their brain weight ratio (BWT). (II) Light microscopy images of the cerebellar cortex of (A) C (–ve) group showing molecular layer (M), granular layer (G), and Purkinje cells (P); (B) C (+ve) group exhibiting disorganized architecture of cerebellar cortex of brain; (C) free MTX group showing inflammation and hemorrhage (black arrows); (D) free-NE COCO group displaying small changes in the cerebellar cortex of brain; and (E) MTX-NE COCO group showing arranged structure of molecular layer, granular layer, and Purkinje cells. Images were magnified at ×40.

*Histology examination*. According to Figure 5(I), the morphologies of the resected brain for the experimental mice were similar as well as their BWT ratios. The brain section of the C (–ve) group (Figure 5(IIA)) displayed the normal brain structure with the normal cerebellar cortex and the normal molecular layer, granular layer, and Purkinje cells. In contrast, the brain tissue structure of the C (+ve) group (Figure 5(IIB)) exhibited disorganized architecture of the cerebellar cortex of the brain and the disappearance of the molecular layer, granular layer, and Purkinje cells. The treated group with free MTX (Figure 5(IIC)) revealed inflammation and hemorrhage in the cerebellar cortex. In contrast, the free-NE COCO treated group (Figure 5(IID)) presented small changes in the cerebellar cortex structure of the brain. Interestingly, MTX-NE COCO (Figure 5(IIE)) exhibited significant improvement in the architecture of the cerebellar cortex.

#### 4.3.2. Lung tissue analysis

**Figure 6.**
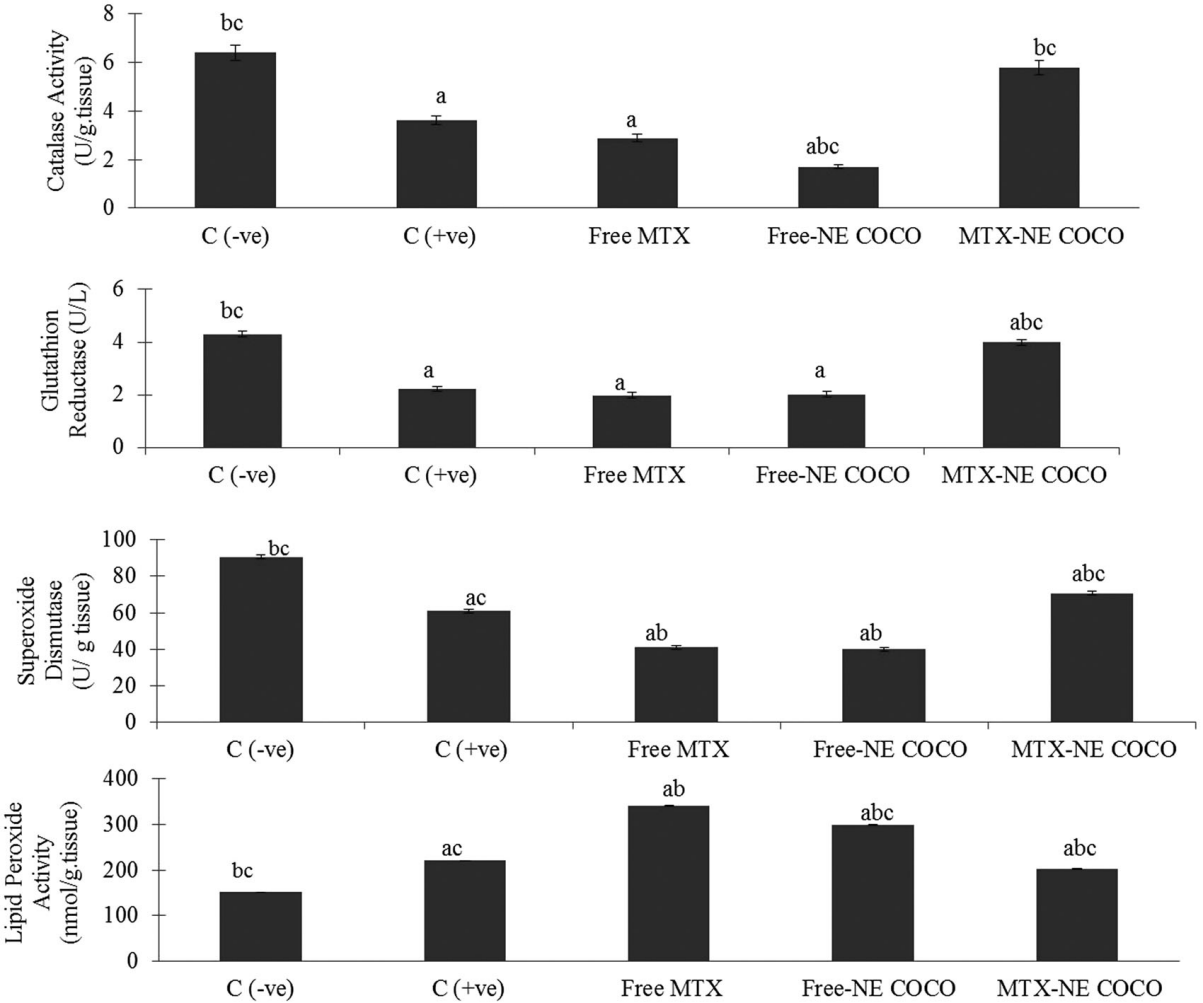
The (ROS) values in lung tissue homogenates of the groups. The superscripts (a, b, c) display the statistical significant variations between the desired group and C (–ve), C (+ve), and free MTX, respectively (*p* < .05).

*Oxidative stress*. The antioxidant status of the lung tissues for all of the tested groups is represented in Figure 6. Among all of the tested groups, all of the antioxidant enzymes activities, CAT, SOD, and GR activities for the C (–ve) group were the greatest while the lipid peroxidation level was the least (*p* <  .05). When compared to the C (+ve) group, it was only the MTX-NE COCO treated mice who got ameliorated oxidative stress since their antioxidant enzyme activities were raised meanwhile their lipid peroxidation were lowered (*p* < .05).

**Figure 7.**
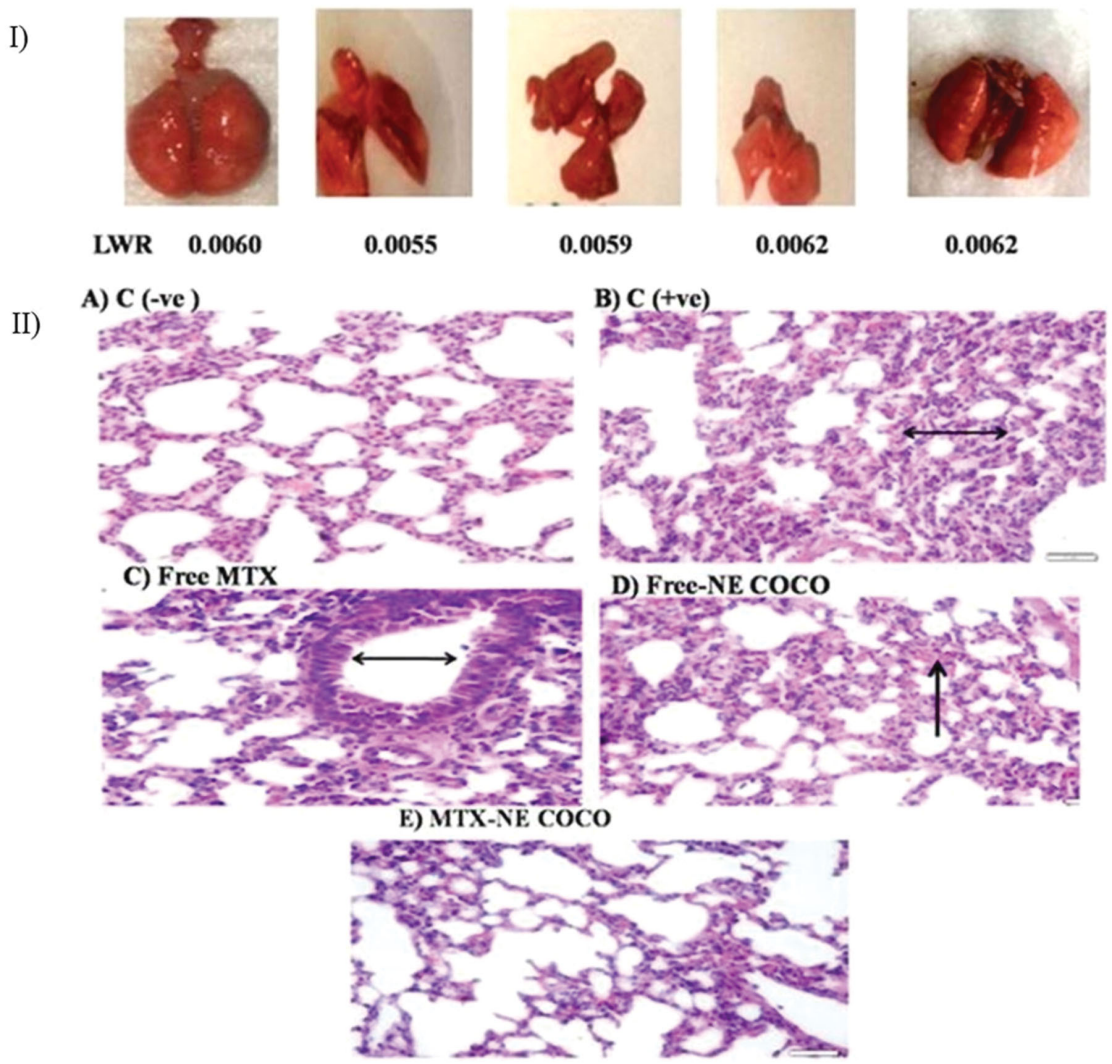
(I) photomicrographs of the resected lung for the tested mice and their lung weight ratio (LWR). (II) Light microscopy images of the lung tissues. (A) C (–ve) group represents the normal tissue of the lung’s mice. (B) C (+ve) group and (C) free MTX group display the abnormality thickness of the alveolar wall (black arrows). (D) The free-NE COCO group exhibits increased hemorrhage (black arrow). (E) MTX-NE COCO group showing the arranged structure of the alveolar wall. Images were magnified at ×40.

*Histology examination*. Although the morphologies of the experimental mice have varied as shown in Figure 7(I), their LWT ratios were comparable. The lung section of the C (–ve) group (Figure 7(IIA)) displayed a normal lung with the thin alveolar wall. In contrast, the lung tissue structure of the C (+ve) group (Figure 7(IIB)) revealed the abnormality thickness of the alveolar wall. The tissue structures of the groups treated with free MTX and free-NE COCO, shown in Figures 7(II(C,D)), have endured hemorrhage and thickening of the alveolar wall. Interestingly, the tissue of the mice treated with MTX-NE COCO (Figure 7(IIE)) exhibited an arranged structure of the alveolar wall.

## 5. Discussion

The present study attempted to solubilize MTX in free-NE COCO with the aim to improve the efficacy of MTX and eliminate its adverse side effects on the brain and lungs. In order to increase the stability of oil-in-water NE, Tween 80 as surfactant and Span 20 as co-surfactant have been used since they are very well known safe pharmaceutical excipients that are used in formulating many drugs (Azmin et al., 1985; Dinarvand et al., 2005; Eskandani et al., 2013; Schwartzberg & Navari, 2018). Azmin et al. (1985) have demonstrated that Tween 80 has ameliorated the pharmacokinetic properties of MTX. The droplet size of the produced free-NE COCO has got enlarged when loaded with MTX which indicates that MTX was located inside the nanodroplet (Dinarvand et al., 2005). Additionally, the magnitude of the negatively charged droplets of free-NE COCO was less than MTX-NE COCO although MTX as a chemical compound has a negative charge at the physiological pH (7.4) (Bryan, 2013; Honary & Zahir, 2013). In spite of the differences among the produced NE formulas in their physical properties, their droplet sizes are still in the nano range implying large surface areas exposed to the cellular membrane and hence enhancement in the cellular uptake of the drugs.

The results of the toxicity screening of the MTT assay have demonstrated that the A549 cells exhibited an increase in their sensitivity when exposed to MTX-NE COCO relative to cells treated with free MTX or free-NE COCO. In addition to the physical properties of the NE delivery in facilitating the permeation of MTX, the inclusion of coconut oil may potentiate the anticancer activity of MTX and eliminate its adverse effects on the lung and brain (Kamalaldin et al., 2015; Rushworth et al., 2015; Koushik et al., 2016; Famurewa et al., 2017; Mahato, 2017). These results are in agreement with previous studies that approved the cytotoxicity of the essential oils and different chemotherapeutic agents were improved when loaded in different nanodelivery systems (Jaiswal et al., 2015; Kamalaldin et al., 2015; Koushik et al., 2016).

For further evaluation of the efficacy and toxicity for the tested drug formulations, they were administered into mice inoculated with the tumor. The present study highlighted the oxidative stress of MTX on the brain and lung which play a major role in the toxicity of MTX (Nishitani Yukuyama et al., 2017). A previous study reported that MTX caused brain injury due to the alteration in the antioxidant activities (Gaies et al., 2012). MTX had induced oxidative stress and DNA damage in the blood tissue besides inflammation and apoptosis in the lung tissue (Saygin et al., 2016). In the present study, the incorporation of MTX into free-NE COCO had eliminated the oxidative stress produced by MTX, which can be attributed to the ability of NE based on coconut oil to improve the efficacy of MTX. It had been demonstrated that coconut oil had antioxidant and protective effects against oxidative stress and the damage induced by MTX in mice (Rushworth et al., 2015; Boateng et al., 2016; Famurewa et al., 2018).

In this study, serum activities of CAT, SOD, and GR levels were remarkably increased in mice treated with free-NE COCO and MTX-NE COCO compared to the free MTX group. These results are in agreement with the previous study that demonstrated the beneficial health effects of coconut oil against oxidative damage and side effects of MTX side effects (Zakaria et al., 2011; Kappally et al., 2015; Famurewa et al., 2017). Recently, several studies have focused on the protective effect of coconut oil against Alzheimer’s disease (Avgerinos et al., 2019; Ota et al., 2019; Chatterjee et al., 2020). They have reported that the medium-chain triglycerides (MCTs) of coconut oil have a potential for the prevention of Alzheimer’s disease because they are easily absorbed, and metabolized in the liver and get converted to Keton’s bodies which are important as an energy source for the brain and might help improve its cognitive function. Additionally, current evidence showed that the MCTs in coconut oil have neuroprotective properties.

## 6. Conclusions

The incorporation of MTX into coconut oil NE in the drug delivery had improved its cytotoxic efficacy against the A549 non-small cell lung cancer cells and eliminated its oxidative stress-induced in the lung and brain. It is recommended to evaluate the antitumor activity of MTX-NE COCO against various cancer cells and examine its adverse side effects on other organs.
